# Gastrodin Induces Ferroptosis of Glioma Cells via Upregulation of Homeobox D10

**DOI:** 10.3390/molecules28248062

**Published:** 2023-12-13

**Authors:** Wenpeng Cao, Jinzhi Lan, Zhirui Zeng, Wenfeng Yu, Shan Lei

**Affiliations:** 1 Department of Anatomy, School of Basic Medicine, Guizhou Medical University, Guiyang 550025, China; caowenpeng@gmc.edu.cn; 2 Key Laboratory of Human Brain Bank for Functions and Diseases, Guizhou Medical University, Guiyang 550025, China; 3 Department of Physiology, School of Basic Medicine, Guizhou Medical University, Guiyang 550025, China; lanjinzhi@gmc.edu.cn (J.L.); zengzhirui@gmc.edu.cn (Z.Z.); 4 Key Laboratory of Endemic and Ethnic Diseases, Ministry of Education, Guizhou Medical University, Guiyang 550025, China; 5 Key Laboratory of Medical Molecular Biology, School of Basic Medicine, Guizhou Medical University, Guiyang 550025, China

**Keywords:** gastrodin, glioma, ferroptosis, proliferation, homeobox D10

## Abstract

Gastrodin, the primary bioactive compound found in Gastrodia elata, has been shown to exhibit neuroprotective properties in a range of neurological disorders. However, the precise mechanisms through which gastrodin influences glioma cells remain unclear, and there is a scarcity of data regarding its specific effects. To ascertain the viability of glioma cell lines LN229, U251, and T98, the CCK-8 assay, a colony formation assay, and a 3D culture model were employed, utilizing varying concentrations of gastrodin (0, 5, 10, and 20 μM). Gastrodin exhibited a notable inhibitory effect on the growth of glioma cells, as evidenced by its ability to suppress colony formation and spheroid formation. Additionally, gastrodin induced ferroptosis in glioma cells, as it can increase the levels of reactive oxygen species (ROS) and peroxidized lipids, and reduced the levels of glutathione. Using a subcutaneous tumor model, gastrodin was found to significantly inhibit the growth of the T98 glioma cell line in vivo. Using high-throughput sequencing, PPI analysis, and RT-qPCR, we successfully identified Homeobox D10 (HOXD10) as the principal target of gastrodin. Gastrodin administration significantly enhanced the expression of HOXD10 in glioma cells. Furthermore, treatment with gastrodin facilitated the transcription of ACSL4 via HOXD10. Notably, the inhibition of HOXD10 expression impeded ferroptosis in the cells, which was subsequently restored upon rescue with gastrodin treatment. Overall, our findings suggest that gastrodin acts as an anti-cancer agent by inducing ferroptosis and inhibiting cell proliferation in HOXD10/ACSL4-dependent pathways. As a prospective treatment for gliomas, gastrodin will hopefully be effective.

## 1. Introduction

Approximately 30% of all tumors affecting the brain and central nervous system are gliomas [1]. Gliomas are highly aggressive and malignant, provoking high morbidity and mortality. Patients with glioblastoma are characterized by increased intracranial pressure, seizures, and cognitive dysfunction. Despite the correlation between glioma incidence and advancing age, nearly half of cases are detected in individuals aged 40–64 [2]. The treatment of glioma remains an unresolved medical challenge, as the current therapeutic approaches, typically involving a combination of surgical resection, radiotherapy, and chemotherapy, fail to yield satisfactory outcomes [3]. In order to combat glioma, new therapeutic drugs and effective therapeutic targets are urgently needed.

The utilization of natural compounds in the therapeutic management of human ailments dates back to the origins of human civilization and serves as the most ancient and extensively employed form of medication owing to their curative attributes [4]. Certain categories of natural compounds hold significant value as reservoirs of efficacious anticancer agents [5]. Gastrodin, a prominent constituent of traditional Chinese medicine, has garnered significant attention due to its extensive pharmacological effects and minimal toxic and adverse reactions [6]. Research has substantiated the efficacy of gastrodin in reducing blood pressure, enhancing blood circulation to the heart and brain, safeguarding nerve cells, retarding central nervous system aging, combating epilepsy, and ameliorating cognitive and emotional impairments in the management of cardiovascular and cerebrovascular ailments [7]. Recent studies have also revealed the promising anti-tumor effects of gastrodin. Liao et al. suggested that gastrodin may serve as a potential antitumor agent by reducing survivin levels in non-small-cell lung cancer [8]. Additionally, Shu et al. demonstrated that gastrodin effectively suppressed the growth of transplanted H22 ascitic hepatic tumor cells in vivo, exhibiting minimal toxicity [9]. The effect of gastrodin on cytotoxicity in human glioma cells remains unclear.

HOXD10 encodes a transcription factor with a sequence-specific homeobox DNA-binding domain that belongs to the Abd-B homeobox family [10]. Research has extensively demonstrated the pivotal role HOXD10 plays during cell differentiation and morphogenesis [11]. Additionally, HOXD10 appears to suppress tumors in a variety of cancers in humans. Notably, Wang et al. conducted a study revealing a significant downregulation of HOXD10 expression in gastric carcinogenesis, which was attributed to promoter hypermethylation [12]. Sun et al. demonstrated that HOXD10 directly modulated the expression of MMP-14 and uPAR, thereby suppressing glioma cell invasion [13]. Therefore, the induction of HOXD10 upregulation is a strategy for glioma treatment.

The purpose of this study was to evaluate the role and key molecular processes of gastrodin in glioma and provide a theoretical basis for glioma treatment. Based on the results, gastrodin upregulated HOXD10, triggered ferroptosis within the analyzed cells, and suppressed glioma cell proliferation. These findings reveal a new mechanism for the anti-glioma activity of gastrodin, which is expected to serve as a novel clinical treatment for glioma.

## 2. Results

### 2.1. Gastrodine Exhibited Prominent Anti-Tumor Effects In Vitro

**Figure 1:**
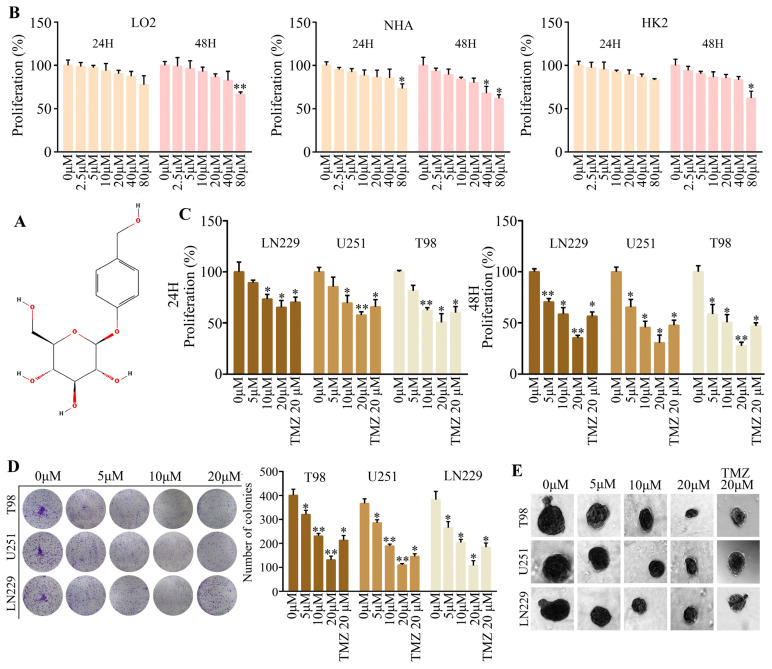
Gastrodin-induced inhibition of glioma cell proliferation in vitro. (**A**) Chemical structure of gastrodin. (**B**) Cell proliferation rates of LO2, HK2, and HNA cells treated with different concentrations (0, 2.5, 5, 10, 20, 40, and 80 μM) of gastrodin after 24 and 48 h as determined using the CCK-8 assay. (**C**) Results of treatment of U251, T98, and LN229 cells with different concentrations (0, 5, 10, and 20 μM) of gastrodin; CCK-8 assay was used to detect proliferation in each group. (**D**) Results of colony formation assay used to detect the colony formation of glioma cells treated with different concentrations (0, 5, 10, and 20 μM) of gastrodin. (**E**) Results of 3D culture model used to detect the spheroid formation of glioma cells treated with different concentrations (0, 5, 10, and 20 μM) of gastrodin. * represents *p* < 0.05; ** represents *p* < 0.01. *n* = 3. The control group was used for comparison. Data are shown as means ± SD.

Gastrodin (Figure 1A) is an active component of Gastrodia elata Blume, chemically known as 4-hydroxybenzyl alcohol-4-O-β-d-glucopyranosid [14]. The treatment of human normal hepatocytes cells (LO2), human renal proximal tubular cells (HK2), and normal human astrocytes (NHA) with different concentrations (0, 2.5, 5, 10, 20, 40, and 80 μM) of gastrodin revealed that 2.5, 5, 10, and 20 μM concentrations of gastrodin were not cytotoxic to LO2, HK2, and NHA after both 24 and 48 h (Figure 1B). To rule out nonspecific cytotoxicity, follow-up experiments were performed using 0, 5, 10, and 20 μM of gastrodin. Further experiments were conducted with LN229, T98, and U251 glioma cells to confirm the effect on proliferation. Compared with the control group, the proliferation rate of the LN229, T98, and U251 cells after gastrodin treatment was significantly reduced. Compared with the 20 μM of temozolomide positive treatment group, the proliferation rate of cells in the 20 μM of gastrodin treatment group was significantly reduced (Figure 1C). Furthermore, the results obtained from the colony formation assays demonstrated a reduction in the number, size, and compactness of colonies in the gastrodin treatment group when compared to those of the control group (Figure 1D). Additionally, inducing the glioma cells’ ability to form cancer spheroids in the ultra-low adsorption microporous plate revealed a significant decrease in the diameter of spheroids following gastrodin treatment (Figure 1E). The results suggested that gastrodin significantly inhibited the proliferation of the glioma cells LN229, T98, and U251, and it was more effective than the positive drug, temozolomide (20 μM).

### 2.2. Gastrodin Induced Glioma Cell Ferroptosis In Vitro

**Figure 2:**
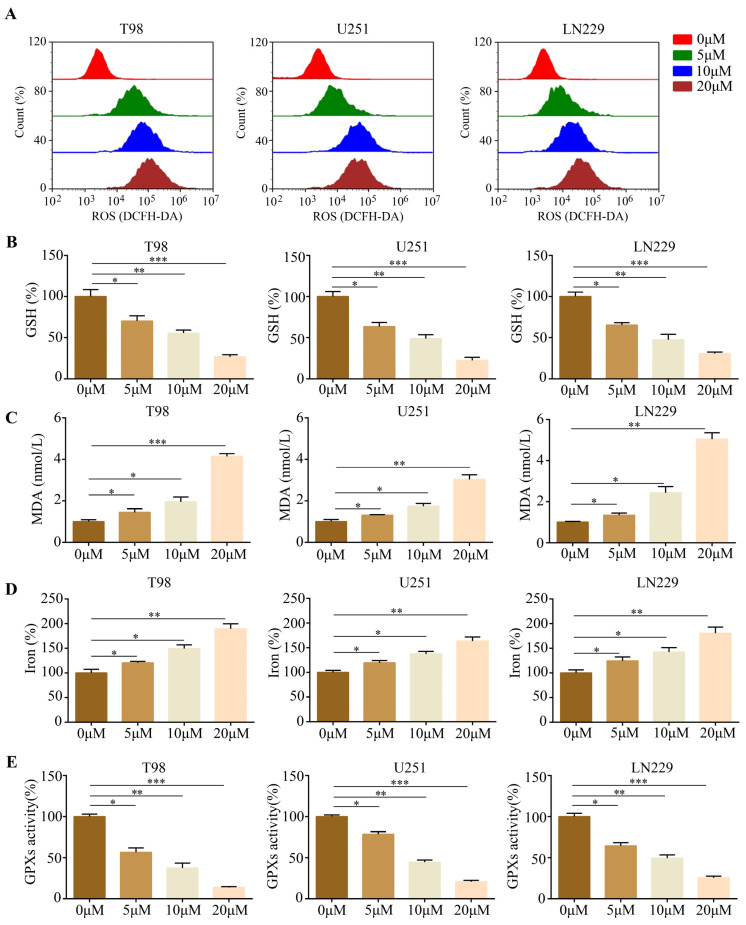
Induction of ferroptosis by gastrodin in glioma cells in vitro. (**A**) Results regarding U251, T98, and LN229 cells treated with different concentrations (0, 5, 10, and 20 μM) of gastrodin; the cellular ROS level was analyzed with a flow cytometer. (**B**) Intracellular GSH levels in glioma cells treated with different concentrations (0, 5, 10, and 20 μM) of gastrodin. (**C**) Intracellular MDA levels in glioma cells treated with different concentrations (0, 5, 10, and 20 μM) of gastrodin. (**D**) Intracellular iron progression in glioma cells treated with different concentrations (0, 5, 10, and 20 μM) of gastrodin. (**E**) Intracellular GPX activities in glioma cells treated with different concentrations (0, 5, 10, and 20 μM) of gastrodin. * represents *p* < 0.05; ** represents *p* < 0.01; *** represents *p* < 0.001. *n* = 5. The control group was used for comparison. Data are shown as means ± SD.

There are several characteristic features of ferroptosis, including reactive oxygen species (ROS), lipid peroxidation, and glutathione depletion [15]. Thus, glioma cells treated with gastrodin produced ROS and glutathione (GSH) as indicators of oxidative stress, in addition to malondialdehyde (MDA). Notably, the glioma cells treated with gastrodin exhibited significantly elevated levels of baseline ROS (Figure 2A). Furthermore, the occurrence of ferroptosis-related processes, including GSH depletion (Figure 2B), MDA production (Figure 2C), and increased iron levels, was observed (Figure 2D). There was a consistent decrease in GPX peroxidase activity in the gliomas treated with gastrodin (Figure 2E). These findings strongly suggest that gastrodin induces ferroptosis in gliomas. 

### 2.3. HOXD10 Was Identified as a Key Target of Gastrodin

**Figure 3:**
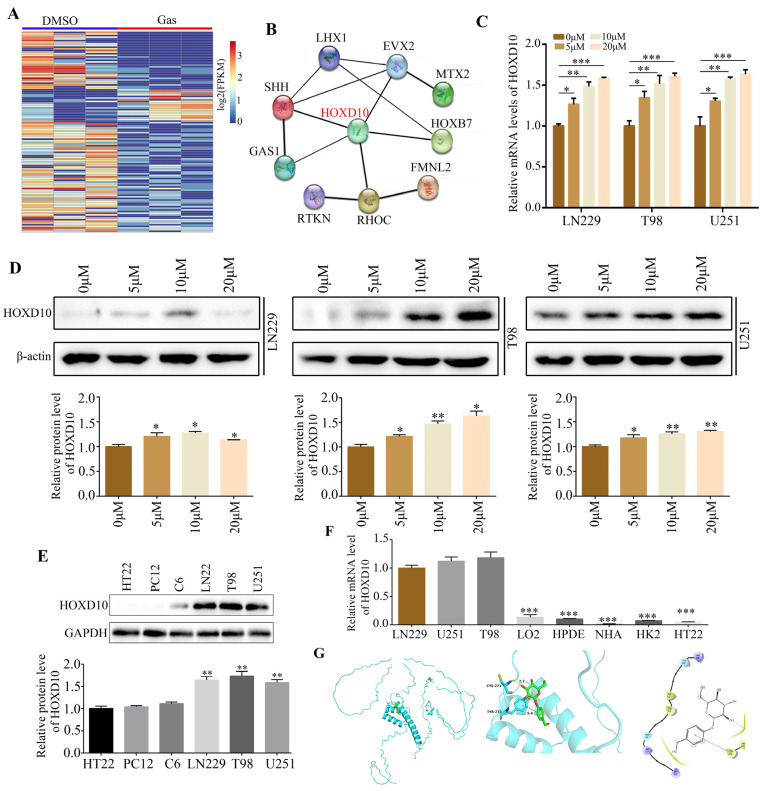
HOXD10 as the key target of gastrodin. (**A**) Identified differentially expressed genes in glioma cells treated with DMSO and gastrodin. (**B**) Results of protein–protein interaction network analysis performed for differentially expressed genes. HOXD10 had strong relationship with other proteins encoded by differentially expressed genes. (**C**,**D**) Results of RT-qPCR and Western blotting performed to analyze the mRNA and protein levels of HOXD10 in glioma cells treated with different concentrations (0, 5, 10, and 20 μM) of gastrodin. (**E**) Results of Western blotting performed to analyze the protein levels of HOXD10 in glioma cells, HT22, C6 and PC12. (**F**) Results of qRT-PCR performed to analyze the mRNA levels of HOXD10 in glioma cells, LO2, HPDE, NHA, HK2, and HT22. (**G**) Binding mode of gastrodin with HOXD10 and 3D illustration of the details of the interaction. Green shows gastrodin; aquamarine shows the HOXD10 protein. * represents *p* < 0.05; ** represents *p* < 0.01; *** represents *p* < 0.001. *n* = 3. The control group was used for comparison. Data are shown as means ± SD.

High-throughput sequencing of gastrodin-treated T98 cells provided a deeper insight into gastrodin’s molecular mechanisms in gliomas. As a result of our analysis, we observed that 102 genes were downregulated and that 20 genes were upregulated (Figure 3A). HOXD10 is strongly associated with other differentially expressed-gene-encoded proteins, as revealed by further analysis of protein–protein interaction networks (Figure 3B). As a result of gastrodin treatment, RT-qPCR and Western blotting experiments revealed that both HOXD10 mRNA and protein levels increased (Figure 3C,D). Interestingly, HOXD10 protein expression levels were higher in the glioma cell lines, including LN229, T98, and U251, relative to HT22, C6 and PC12 (Figure 3E). Furthermore, the HOXD10 mRNA expression level was lower in the normal cells LO2, HPDE, NHA, HK2, and HT22 compared with that in the glioma cell lines LN229, T98, and U251 (Figure 3F). An analysis of gastrodin-HOXD10 protein binding was then performed using molecular docking technology. The 3D image shows gastrodin stabilizing HOXD10 by binding to CYS271 and TYR273 (Figure 3G). 

### 2.4. ACSL4 Is a Downstream Gene of HOXD10

**Figure 4:**
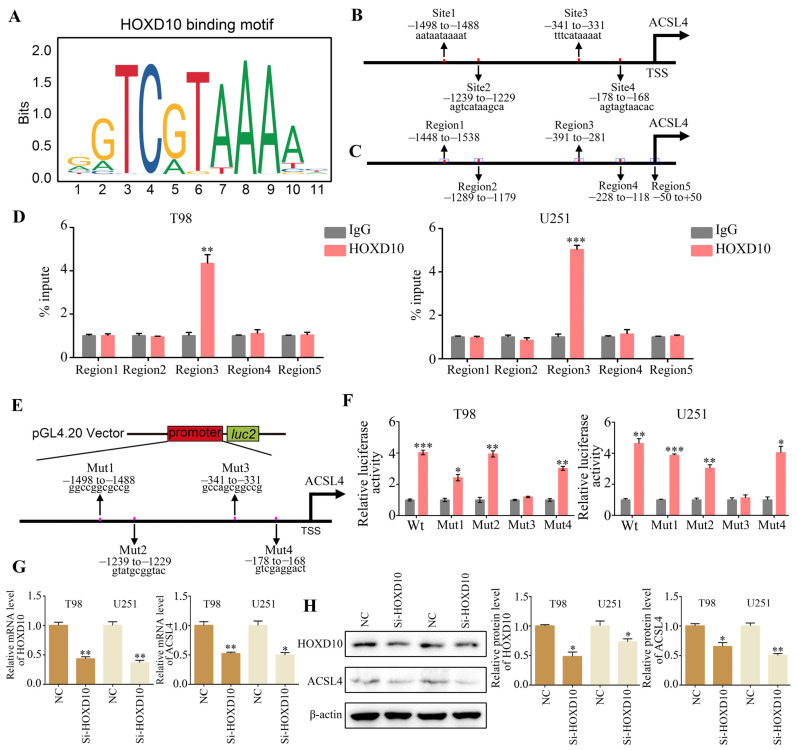
HOXD10 activates the transcription of ASCL4. (**A**) HOXD10 binding motif predicted via JASPAR. (**B**) Schematic diagram of potential HOXD10 binding sites in the promoter region of ASCL4. (**C**) Schematic diagram of primers designed for regions of ASCL4 promoter. (**D**) ChIP-PCR analysis of enrichment of HOXD10 on ASCL4 promoter. IgG was used as a negative control. (**E**) Schematic diagram of dual-luciferase reporter vector construction for ASCL4 promoter. (**F**) The luciferase activity of the wild-type and mutant ASCL4 promoters with indicated cells. (**G**,**H**) The results of RT-qPCR and Western blotting used to detect the effect of HOXD10 knockdown on the mRNA and protein expression of ASCL4. * represents *p* < 0.05; ** represents *p* < 0.01; *** represents *p* < 0.001. *n* = 3. The control group was used for comparison. Data are shown as mean ± SD.

Transcriptional factors are able to interact with specific gene promoter sequences, thereby controlling downstream gene transcription and functional activation. Using the JASPAR database to obtain the HOXD10 binding sequences, we conducted a comparison between the ACSL4 promoter region sequences and the HOXD10 binding sequences (Figure 4A). As a result of this analysis, it was determined that ASCL4 contains four potential HOXD10 binding sites (Figure 4B). Afterward, we created PCR primers that target the estimated 100 bp region containing the presumed binding locations and sites where transcription begins (Figure 4C). The ChIP-PCR findings demonstrated that the amplification of the binding sequence between the ASCL4 promoter and HOXD10 was facilitated by DNA precipitated by Flag-tagged HOXD10, implying a direct binding of HOXD10 to the ASCL4 promoter regions (Figure 4D). In order to delve deeper into the transcriptional activity of HOXD10, the promoter sequences containing the wild type (Wt) and each possible binding site mutant (Mut) were inserted into the pGL4.20 plasmid and transfected into HOXD10 overexpression and control glioma cells (Figure 4E). The luciferase assay demonstrated that the overexpression of HOXD10 led to an increase in the activity of the ASCL4 promoter. However, no significant effect was observed when the HOXD10 binding site was mutated (Figure 4F). This suggests that HOXD10 may facilitate the transcription of ACSL4 by directly binding to its promoter regions. Furthermore, RT-qPCR and Western blot analysis revealed that the enhanced transfection with HOXD10 siRNAs resulted in a decrease in both ASCL4 mRNA and protein expression in glioma cells (Figure 4G,H).

### 2.5. Silencing of HOXD10 Attenuated the Promotion of Ferroptosis by Gastrodin in Glioma Cells 

**Figure 5:**
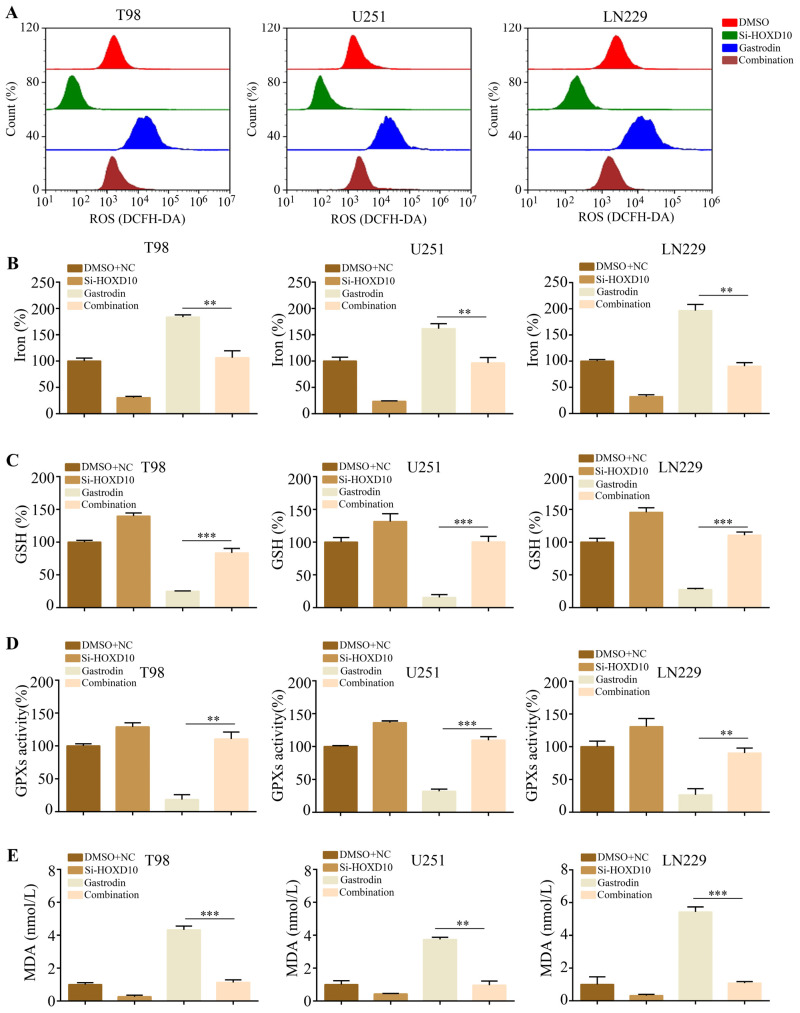
Silencing of HOXD10 reversed the promotive effect of gastrodin on ferroptosis in glioma cells. Glioma cells were treated with DMSO + NC siRNA, gastrodin, HOXD10 siRNAs, and gastrodin + HOXD10 siRNAs, respectively. (**A**) The cellular ROS level was analyzed using a flow cytometer in each group. (**B**) Intracellular iron progression in glioma cells in each group. (**C**) Intracellular GSH levels in glioma cells in each group. (**D**) Intracellular GPX activities in glioma cells in each group. (**E**) Intracellular MDA levels in glioma cells in each group. ** represents *p* < 0.01; *** represents *p* < 0.001. *n* = 3. The control group was used for comparison. Data are shown as mean ± SD.

HOXD10 has been identified as a suppressor in multiple cancer types. Consequently, we postulated that HOXD10 plays a role in the biological processes induced by gastrodin. To investigate this, we introduced HOXD10 siRNAs into glioma cells prior to administering the gastrodin treatment. Notably, the glioma cells exhibited an augmentation of ferroptosis-related phenomena following gastrodin treatment; elevated baseline reactive oxygen species (ROS) levels (Figure 5A), including an elevation in iron levels (Figure 5B); a reduction in GSH levels (Figure 5C); a decline in GPX activity (Figure 5D); and an increase in MDA production (Figure 5E). The silencing of HOXD10 attenuated the above effects. 

### 2.6. Silencing of HOXD10 Attenuated the Inhibitory Effect of Gastrodin on Glioma Cell Proliferation

**Figure 6:**
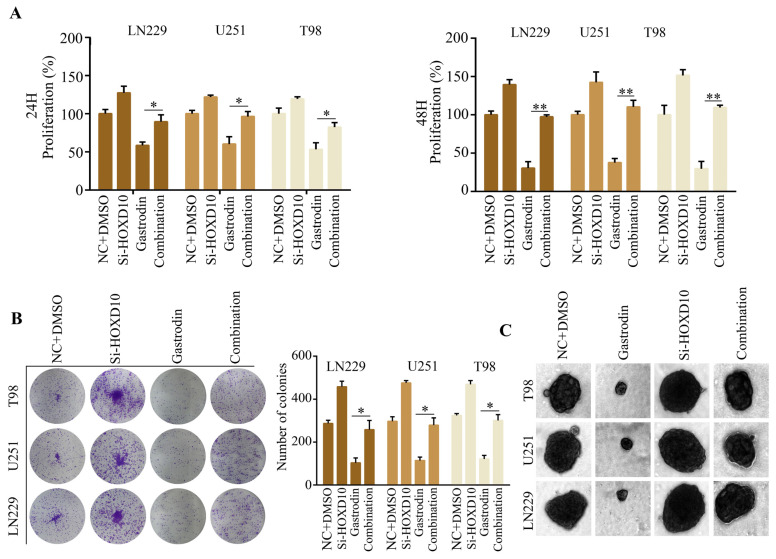
Silencing of HOXD10 reversed the promotive effect of gastrodin on ferroptosis in glioma cells. Glioma cells were treated with DMSO + NC siRNA, gastrodin, HOXD10 siRNAs, and gastrodin + HOXD10 siRNAs, respectively. (**A**) Results of the use of CCK-8 to detect the proliferative rates of glioma cells in each group. (**B**) Results of colony formation assay used to detect the colony formation of glioma cells in each group. (**C**) Results of 3D culture model used to detect the sphere formation of glioma cells in each group. * represents *p* < 0.05; ** represents *p* < 0.01. *n* = 3. The control group was used for comparison. Data are shown as mean ± SD.

Based on the findings obtained from the CCK-8 assay, it was observed that the inhibition of HOXD10 effectively mitigated the 24 and 48 h suppression of glioma cell proliferation induced by gastrodin (Figure 6A). Furthermore, the colony formation assays revealed that the silencing of HOXD10 counteracted the suppressive impact of gastrodin on the formation of colonies in glioma cells (Figure 6B). Similarly, the 3D culture model demonstrated that the silencing of HOXD10 alleviated the suppressive effect of gastrodin on the diameters of spheres formed by glioma cells (Figure 6C).

### 2.7. Gastrodin Suppressed the Proliferation of Glioma Cells In Vivo

**Figure 7:**
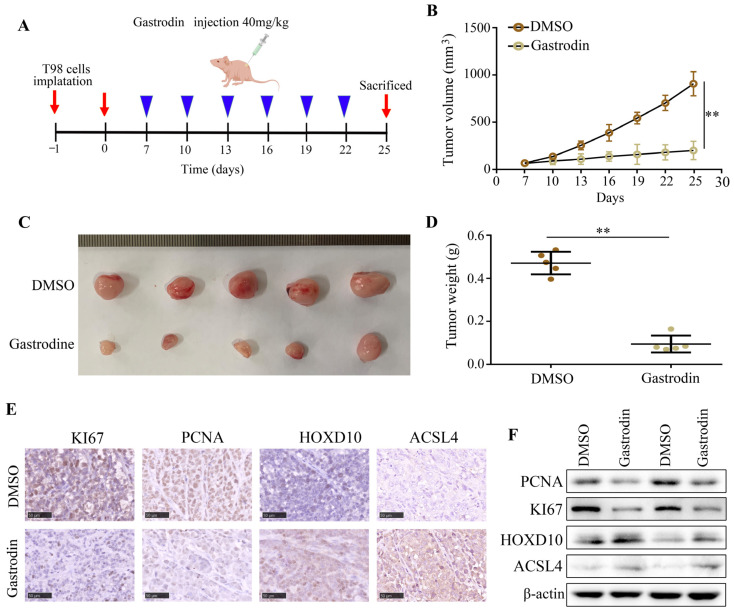
Gastrodin repressed the proliferation rate of T98 cells in vivo and increased the expression of HOXD10. (**A**) Schematic representation of animal experiments. (**B**,**C**) Proliferation of tumor tissues in the DMSO and gastrodin treatment groups. (**D**) Tumor weight in the DMSO and gastrodin treatment groups. (**E**) Expression of PANC, KI67, HOXD10, and ACSL4 in tumor tissues in the DMSO and gastrodin treatment groups. Black lines in the bottom left corner indicate 50 μm. (**F**) Using Western blotting, the expression of PANC, KI67, HOXD10, and ACSL4 was detected in tumor tissues in the DMSO and gastrodin treatment groups. ** represents *p* < 0.01. *n* = 3. The control group was used for comparison. Data are shown as mean ± SD.

The in vivo efficacy of gastrodin treatment was evaluated by subcutaneously administering T98 cells to BALB/c nude mice. The experimental groups were randomly divided into DMSO and gastrodin (40 mg/kg) groups. In the DMSO group, tumor growth was observed to be rapid, whereas gastrodin treatment significantly suppressed tumor growth (Figure 7A–C) and reduced tumor weight (Figure 7D). Moreover, the gastrodin treatment led to decreased levels of KI67 and PCNA protein expression and increased levels of HOXD10 and ACSL4 in the tumor tissues compared to the DMSO treatment (Figure 7E,F). These findings demonstrate the evident anti-tumor effect of gastrodin in an in vivo setting.

## 3. Discussion

In recent years, Traditional Chinese Medicine (TCM) has garnered significant interest from researchers globally due to its distinctive merits, encompassing diverse pathways, multiple targets, minimal toxicity, and limited adverse effects [16]. Gastrodia elata Blume, a renowned herb prevalent throughout Asia, has been employed in medicinal practices for millennia in Korea, China, and Japan [17]. Gastrodia elata Blume contains various phenolic compounds, such as gastrodin, vanillin, 1,3-bis(4-hydroxybenzyl)citrate, 1-(4-beta-D-glucopyranosyloxybenzyl) citrate, parishin B, 4-hydroxybenzyl alcohol (4-HBA), and 4-hydroxybenzaldehyde (4-HBZ) [18]. Previous studies have demonstrated that gastrodin possesses anticonvulsant, analgesic, sedative, free-radical-scavenging, and anti-anxiety properties, which effectively alleviate symptoms associated with headaches, epilepsy, dizziness, rheumatism, neuralgia, paralysis, hypertension, and other neuralgic disorders [19,20]. Liang et al. demonstrated that gastrodin inhibited glioma cell proliferation by inducing oxidative-stress-related apoptosis, cell cycle arrest, and p53 activation [21]. However, the potential effect of gastrodin on gliomas remains unexplored both domestically and internationally.

In the current investigation, a sequence of functional experiments was conducted to ascertain the impact of gastrodin on glioma cell proliferation, colony formation, and ferroptosis induction. We found that gastrodin significantly inhibited glioma cell proliferation and colony formation as well as induced ferroptosis. However, researchers have demonstrated that gastrodin suppressed ferroptosis via the regulation of the Nrf2 in HT-22 cells [22,23]. Studies indicate that gastrodin inhibits H_2_O_2_-induced ferroptosis through its antioxidative effect in the rat glioma cell lines C6 and PC12 [24]. In the present study, RNA-seq analysis was employed to explore the molecular profile and biological role of gastrodin in glioma cells, revealing significant alterations in 122 genes subsequent to gastrodin treatment. HOXD10 was significantly upregulated after treatment with gastrodin in the gliomas. Notably, gastrodin significantly upregulated HOXD10 expression in human glioma cells compared with the HT22, PC12, LO2, HPDE, NHA, HK2, and C6 cells. These results indicate that gastrodin binds to its target HOXD10 to promote ferroptosis in gliomas.

HOX genes are responsible for encoding proteins that serve as pivotal master regulatory transcription factors during embryogenesis and are also implicated in the progression of cancer [25,26]. In humans, these genes are organized into four clusters (HOX-A, HOX-B, HOX-C, and HOX-D) located on distinct chromosomes [27]. Currently, the expression of HOX family genes has been documented in various tumor types, including lung cancer, breast cancer, and gliomas [28,29,30]. HOXD10 has been widely acknowledged as a tumor suppressor and has been observed to be downregulated in various cancer types, including colon carcinomas, early-stage head and neck squamous cell carcinoma, and oral squamous cell carcinoma [31,32]. In the present study, we found that the HOXD10 binding site promotes ACSL4 transcription by interacting with the ACSL4 promoter specifically.

Ferroptosis is a regulated mechanism of cellular demise that occurs due to the buildup of membrane lipid peroxides resulting from an excess of iron [33]. Long-chain acyl-CoA synthetase-4 (ACSL4), which belongs to the long-chain acyl-coenzyme synthetase (ACSL) family, plays a crucial role in the synthesis and breakdown of fatty acids [34]. The disruption of ACSL4 function is linked to various disorders related to lipid metabolism [35]. In contrast to other members of the family, ACSL4 serves as a catalyst for the conversion of arachidonic acid (AA) to arachidonic acid coenzyme A, the latter of which is involved in the synthesis of phospholipids in cell membranes [36]. ACSL4 is responsible for the enrichment of long polyunsaturated ω6 fatty acids in cell membranes, thereby influencing the sensitivity of ferroptosis by modulating cellular lipid composition [37]. The present study conducted by Hua demonstrates the pivotal role of ACSL4 in promoting erastin-induced ferroptosis through lipotoxicity mediated by 5-HETE [38]. 

In the current investigation, it was observed that gastrodin exhibited inhibitory effects on the proliferation of glioma cells and induced ferroptosis. Furthermore, the administration of gastrodin resulted in an elevation in HOXD10 mRNA levels within the glioma cells. Notably, HOXD10 demonstrated a specific interaction with the ACSL4 promoter, leading to an upregulation of ACSL4 expression. The subsequent knockdown of HOXD10 significantly attenuated the impact of gastrodin on glioma cell proliferation and ferroptosis. These findings strongly suggest the involvement of HOXD10 in the biological processes induced by gastrodin. However, the specific anti-glioma mechanism of gastrodin is complicated. Our study only considered HOXD10 as an example to study.

## 4. Materials and Methods

### 4.1. Cell Culture

Immortalized mouse hippocampal cells (HT22), rat glioma cells (C6), human normal hepatocytes cells (LO2), human renal proximal tubular cells (HK2), human pancreatic duct epithelial cells (HPDE), normal human astrocytes (NHA), and human glioma cell lines (U251, T98, and LN229) were obtained from the American Type Culture Collection (ATCC, USA). Pheochromocytoma (PC12) cells were obtained from Pricella (Wuhan, China). The cells were cultured in Dulbecco’s modified Eagle’s medium (DMEM; Gibco, USA) containing 10% fetal bovine serum (BI, Kibbutz Beit Haemek, Israel) at 37 °C with 5% CO_2_. Gastrodin and temozolomide powder (MCE, Wuhan, China) were mixed with DMSO to form a 10 mol·L^−1^ mother liquor, which was then filtered, sterilized, and stored at −20 °C. The working solution was diluted to various concentrations in the DMEM culture solution. The HOXD10 siRNAs were constructed (Invitrogen, Carlsbad, CA, USA) and transfected into cells using Lipofectamine 2000 (Thermo Fisher Scientific, Waltham, MA, USA). 

### 4.2. CCK-8 Assay

Glioma cells in the logarithmic growth phase were subjected to trypsin digestion to produce a cell suspension. Subsequently, 3 × 10^3^ cells were seeded in each well of a 96-well plate, with six holes being punctured in each group. Following a 48 h treatment period, the medium in each well was replaced with 90 μL of serum-free DMEM solution mixed with 10 μL of CCK-8 solution. The cells were then incubated for 2 h; subsequently, the absorbance of each well at 450 nm was measured using a microplate reader.

### 4.3. Spheroid Formation Experiment

Cells were collected and resuspended in DMEM containing 10% FBS. Then, the cells were seeded onto a 96-well ultra-low adsorption microporous plate (Corning, NY, USA) at a density of 1000 cells/well. After spheroid formation, the change in the diameter of the spheroids was detected every two days until day 12.

### 4.4. Computer Molecular Docking

The structure of gastrodin was obtained from PubMed Compound (https://www.ncbi.nlm.nih.gov/pccompound (accessed on 8 August 2023)). The crystal structures of HOXD10 were obtained from the Protein Data Bank (https://www.rcsb.org/ (accessed on 8 August 2023)). Then, gastrodin and target structures were imported to the Autodock software (version 4.2) to perform flexible docking according to the standard procedure. The binding sites of the targets and gastrodin were visualized using Pymol software (version 1.1).

### 4.5. RNA Sequencing

TRIzol reagent was used to extract total RNA from samples. A Bioanalyzer 2100 and RNA 6000 Nano LabChip Kit (Agilent, CA, USA) were used to determine the RNA quantity and purity, and RNA samples with RNA integrity numbers > 7.0 were used to create sequencing libraries. Reverse transcription was performed using SuperScriptTM II Reverse Transcriptase (Invitrogen, Carlsbad, CA, USA), and the mRNA was fragmented into short pieces following purification. The cDNA inserts averaged 300 ± 50 base pairs for the final library. The cDNA was sequenced using an Illumina NovaseqTM 6000 (LC-Bio Technology, Ltd., Hangzhou, China) in accordance with the vendor’s protocol. After high-quality clean reads were obtained and batch normalization was performed, the count data were analyzed using the EdgeR package. *p* values < 0.05 and |LogFold-Change| values ≥ 2 were used as test criteria for differentially expressed genes.

### 4.6. Subcutaneous Tumorigenesis Experiments

A suspension of T98 cells (2 × 10^6^ cells per 200 μL) was injected subcutaneously into the armpit of the right forelimb of each nude mouse. On the seventh day, vernier calipers were used to determine the length and width of each subcutaneous tumor, and tumor volume (mm^3^) was calculated as follows: (length × width^2^)/2. Mice with tumors measuring 40–60 mm^3^ were retained for further studies, in which they were intraperitoneally injected every three days with DMSO or gastrodin (40 mg/kg/day). The tumor volume was determined every three days and used to plot the tumor growth curve. After 25 days of treatment, the mice in each group were sacrificed, their tumor tissues were removed, and their tumor weights were measured.

### 4.7. Histology and Immunohistochemistry

Total RNA was extracted from samples using the TRIzol reagent (Thermo Fisher Scientific, Waltham, MA, USA). Immunohistochemistry was performed on the tumor tissues. After permeabilization, dehydration, and antigen retrieval, 0.3% H_2_O_2_ and 5% BSA were used to block the tumor sections. Then, the tumor sections were stained with antibodies against HOXD10 (1:100, Cat no. A23992, ABclonal, Wuhan, China), ASCL4 (1:100, Cat no. A14439, Abconal, Wuhan, China), KI67 (1:200, Cat no. 27309-1-AP, Proteintech, Wuhan, China), and PCNA (1:200, Cat no. 10205-2-AP, Proteintech, Wuhan, China) for 12 h at 4 °C. After being washed with PBS three times, horseradish-peroxidase-conjugated secondary antibodies were used to stain sections, and 3,3-diaminobenzidine was stained to detect the immunologic signals. After using hematoxylin to stain the nucleus, the tumor sections were imaged using a light orthophoto microscope (magnification ×200, Olympus, Tokyo, Japan).

### 4.8. Measurement of Cellular Ferroptosis Levels

Solarbio (Beijing, China) provided a glutathione assay kit and instructions for measuring glutathione. Solarbio’s lipid peroxidation assay kit (Beijing, China) was used to determine MDA concentrations. In accordance with the manufacturer’s instructions, iron concentration was measured using a solarbio iron assay kit (Beijing, China). A GPXs Assay Kit (Solarbio, Beijing, China) was used to quantify relative GPX activity. 

### 4.9. Statistical Analysis

All results were analyzed using SPSS software (version 19.0). Student’s t-test was used to analyze the difference between two groups, while one-way analysis of variance combined with the least significant difference t-test was used to compare multiple groups. The significance threshold was set at *p* < 0.05.

## Data Availability

Data are contained within the article.

## Abbreviations

HOXD10	Homeobox D10
ROS	Reactive oxygen species
MDA	Malondialdehyde
GSH	Glutathione
ACSL4	Acyl-CoA synthetase long-chain family member 4
RT-qPCR	Real-time quantitative polymerase chain reaction
